# TGF–β3 Loaded Electrospun Polycaprolacton Fibre Scaffolds for Rotator Cuff Tear Repair: An in Vivo Study in Rats

**DOI:** 10.3390/ijms21031046

**Published:** 2020-02-05

**Authors:** Janin Reifenrath, Mathias Wellmann, Merle Kempfert, Nina Angrisani, Bastian Welke, Sarah Gniesmer, Andreas Kampmann, Henning Menzel, Elmar Willbold

**Affiliations:** 1 Department of Orthopaedic Surgery, Hannover Medical School, Anna–von–Borries Str. 1–3, 30625 Hannover, Germany; 2 Lower Saxony Centre for Biomedical Engineering, Implant Research and Development (NIFE), Hannover Medical School, Stadtfelddamm 34, 30625 Hannover, Germany; 3 Laboratory for Biomechanics and Biomaterials, Department of Orthopaedic Surgery, Hannover Medical School, Haubergstraße 3, 30625 Hannover, Germany; 4 Clinic for Cranio–Maxillo–Facial Surgery, Hannover Medical School, Carl–Neuberg–Straße 1, 30625 Hannover, Germany; 5 Institute for Technical Chemistry, Braunschweig University of Technology, Hagenring 30, 38106 Braunschweig, Germany

**Keywords:** tendon defect, rotator tear cuff, animal study, PCL, implant, protein loading, rat model

## Abstract

Biological factors such as TGF–β3 are possible supporters of the healing process in chronic rotator cuff tears. In the present study, electrospun chitosan coated polycaprolacton (CS–g–PCL) fibre scaffolds were loaded with TGF–β3 and their effect on tendon healing was compared biomechanically and histologically to unloaded fibre scaffolds in a chronic tendon defect rat model. The biomechanical analysis revealed that tendon–bone constructs with unloaded scaffolds had significantly lower values for maximum force compared to native tendons. Tendon-bone constructs with TGF–β3-loaded fibre scaffolds showed only slightly lower values. In histological evaluation minor differences could be observed. Both groups showed advanced fibre scaffold degradation driven partly by foreign body giant cell accumulation and high cellular numbers in the reconstructed area. Normal levels of neutrophils indicate that present mast cells mediated rather phagocytosis than inflammation. Fibrosis as sign of foreign body encapsulation and scar formation was only minorly present. In conclusion, TGF–β3-loading of electrospun PCL fibre scaffolds resulted in more robust constructs without causing significant advantages on a cellular level. A deeper investigation with special focus on macrophages and foreign body giant cells interactions is one of the major foci in further investigations.

## 1. Introduction

In recent decades, remarkable progress in different fields of medicine was achieved with respect to e.g., diagnostic tools, pharmacological and therapeutic approaches, or surgical techniques. One basis for this progress is the insight that a comprehensive understanding into the underlying molecular and physiological mechanisms of diseases and/or healing processes is necessary to understand the causes and processes of any kind of pathophysiological changes. Especially with respect to trauma or orthopaedic surgery, where non-biological implant materials such as screws, plates or whole joint replacements play an important role, a close interdisciplinary cooperation between surgeons, life and materials sciences is indispensable. For many orthopaedic problems, established surgical and therapeutic techniques are present, but complex and chronic diseases especially with respect to tendon and enthesis repair are still a challenge [1]. Chronic rotator cuff tears are among the most observed clinical problems of the upper extremity [2,3]. These tears arise slowly and it often takes a longer time span before affected patients seek medical attention. However, during this time span, unfavourable pathological changes such as the retraction of the tendon, muscle atrophy, and fatty infiltration can occur [4] and these chronic degenerations lead to poor healing results not only in humans but also in animals [5]. This means that surgeons often face a wide range of very complex and confusing damage patterns which make it difficult to choose and apply optimal treatments. Multiple factors are responsible for these differences and complications, including increased age of the patients, different grades of tendon quality, muscle atrophy, presence of scar and granulation tissue or tear size. Although different surgical techniques and therapy strategies are in clinical use [6,7], rotator cuff surgeries in general are often associated with remarkable post-operative complications in terms of re-tears (11% up to 94%, [8]). Even without any post-operative complications, a reduced strength of the healed tendon compared to healthy tendons is often observed [9,10]. To approach these problems, during recent years attention has moved away from rather technical–surgical aspects, e.g., increasing the tensile strength of sutures, towards a better understanding of the biological fundamentals and the mechanical, molecular and cellular mechanisms of tendon healing and especially the biological characteristics of the enthesis and the tendon-to-bone environment [11,12,13,14].

A promising approach to overcome the complex problems of rotator cuff tear repair could be the support of natural healing processes using specialised biodegradable grafts [15,16,17,18]. Promising candidates for such grafts are electrospun fibre scaffolds consisting of polycaprolactone (PCL; [19,20]. These scaffolds consist of spatially oriented fibres with a very high surface area which is a prerequisite for the possible binding of biologically active molecules and the binding of cells [21,22]. PCL is biodegradable with slow degradation rates in vivo [23,24] and PCL-based materials for medical devices such as Capronor, a subdermal implant for long-term drug delivery [25] or drug delivery systems for glaucoma treatment [26] are already in clinical use or trial. Studies show that surface modification with fibronectin or blending with polylactide acid (PLA) improve cell attachment of endothelial cells or cell infiltration with trophoblast cells [27,28] as well as osteogenic and tenogenic differentiation of C3H10T1/2 pluripotent stem cells [29]. Furthermore, PCL–PLA and PCL–gelatine fibre mats seeded with mesenchymal stem cells showed high biocompatibility with viabilities higher than 82% after seven days as reported by Gryshkov and coworkers [30]. A disadvantage of PCL is its uniform molecular structure and its hydrophobic surface which both complicates the chemical binding of molecules and hinders the attachment of cells in vivo [31]. However, both negative effects could be successfully addressed by the use of a special cationic amino group bearing chitosan surface modification (CS–g–PCL). This surface modification both increased wettability and cell attachment and moreover allowed the direct binding of different drug delivery systems or biologically active molecules [32]. It has already been shown that this surface modification has positive effects onto cellular attachment in vitro [32]. Progressing graft vascularisation could be monitored when compared to PCL fibres without such a surface modification in vivo in a special femur chamber model [33]. Due to these studies [32,33], CS–g–PCL fibre scaffolds are an interesting starting material for the delivery of biologically active molecules in rotator cuff tear repair. Furthermore, the oriented arrangement of the fibres inside the scaffolds may serve as a possible guiding structure for regenerating tendon fibres but especially for ingrowing blood vessels and immigrating cells with regenerative capacities which both are the basis for the development of a successfully regenerated tendon–bone-transition [34].

With respect to the tendon-to-bone environment and especially to the enthesis, members of the transforming growth factor β (TGF–β) superfamily are of particular interest because these proteins are known to stimulate chondrogenic differentiation of mesenchymal stromal cells [35,36,37,38,39,40]. Regarding the healing of rotator cuff tears it has already been shown that the sustained delivery of TGF–β3 accelerated the healing process. In vitro, human bone marrow stem cells showed a tenogenic commitment when cultured on aligned chitosan–PCL nanofibres [41] and in vivo, an increase in inflammation, cellularity, vascularity, and cell proliferation, together led to significant improvements in structural properties [42]. Histologically, an increased fibrocartilage and improved collagen organization and biomechanically, a significant higher load at failure could be observed [43].

The aim of the present study was to investigate the effect of an additional TGF–β3–loading on new developed CS–g–PCL fibre scaffolds which were already tested in a former study with respect to biomechanical and histological parameters using an established chronic tendon defect model [44]. The hypothesis of this study was that the use of TGF–β3 loaded fibre scaffolds can support and improve rotator cuff healing after a chronic rotator cuff defect in a small animal model and might be used as base material in the process of the development of implant materials with graded loading of active biological substances.

## 2. Results

### 2.1. Clinical Observations

In the TGF–β3–CS–g–PCL group one animal dropped out of the analysis due to sudden death in the second surgery. All other animals showed normal post-operative follow up with no abnormalities in general condition, wound healing or spontaneous behaviour.

Lameness after the second surgery was compared between the TGF–β3–CS–g–PCL group and the CS–g–PCL control group. At day three, the median score for lameness was already one in the CS–g–PCL group (median 1; minimum 1; maximum 5) whereas in the TGF–β3–CS–g–PCL– group it was two (median 2; minimum 1; maximum 5) and reached score value one at day four. In both groups the median score value zero (median 0; minimum 0; maximum 1) was reached at day eight. In the CS–g–PCL group one animal was slightly lame until day nine and in the TGF–β3–CS–g–PCL group three animals showed a slight lameness until day nine and two animals until day ten.

Weight loss with more than 10% occurred only in one animal during the study and was caused by a misalignment of the incisors. After cutting the incisors, weight increased again. All other animals showed a maximum of 5% weight loss after the surgical procedures and all animals reached or exceeded their initial weight at the end of the study period.

### 2.2. Macroscopical Appearance of Tendon-to-Bone Interface

**Figure 1:**
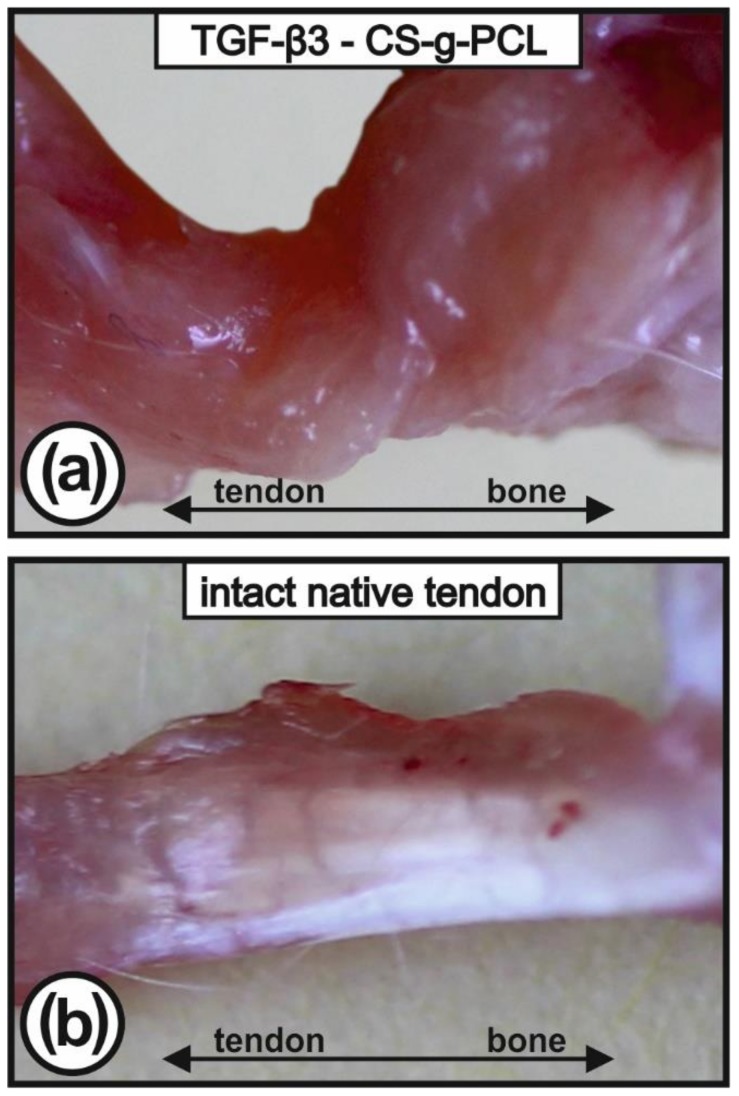
Macroscopic view of the explanted infraspinatus tendons (surgical site with TGF–β3 – CS–g–PCL scaffold (**a**); intact native tendon (**b**)). The left tendons are ensheathed by repair tissue which was mostly left untouched to avoid any mechanical lesion of the construct.

The reconstructed tendons of the left shoulder were ensheathed with a red appearing tissue, consisting of a mixture of tendinous, fibrous, muscular, and especially granulation tissue (Figure 1a), whereas on the contralateral native side the shiny white tendons could be easily recognised (Figure 1b).

### 2.3. Tendon Healing in Rats: Biomechanical Testing

**Figure 2:**
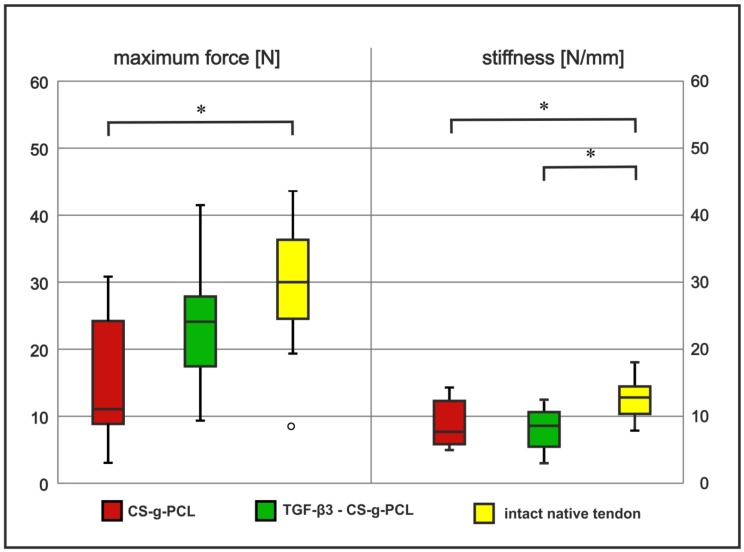
Differences in median values of maximum forces (load to failure; left columns) and in stiffness (right columns) are shown for groups with refixed tendons (with CS–g–PCL scaffold [red bars] and TGF–β3–CS–g–PCL scaffold [green bars]) compared to the intact native tendon [yellow bars]. Significant differences (significance level of α = 0.05) are marked with asterisk (*).

The tendon–bone entheses (*n* = 11; TGF–β3–CS–g–PCL group) were examined biomechanically and compared to the results of the CS–g–PCL control group (*n* = 12). Maximum force values were normalized to the weight of the donors prior to statistical evaluation (Figure 2). Whereas the tendon–bone entheses of the native contralateral side reached values of 29.6 N ± 7.9 N, the tendon–bone constructs of the CS–g–PCL group showed significantly lower values of 15.7 N ± 9.4 N (*p* ≤ 0.01). The tendon–bone constructs of the TGF–β3–CS–g–PCL group were higher than values of the CS–g–PCL group and slightly lower than that of the intact native tendon (23.2 N ± 9.4 N) but not significantly. There was no difference in stiffness of the repaired tendon between CS–g–PCL group and TGF–β3–CS–g–PCL group. Compared to the native control (12.46 ± 0.55 N/mm) the TGF–β3–CS–g–PCL group (8.06 ± 0.92 N/mm) as well as the CS–g–PCL group (7.74 ± 0.91 N/mm) showed significant lower stiffness values (*p* ≤ 0.01). Comparing the areas of construct failure, 61% of the tendons in the native contralateral side group failed in the proximal area near the tendon–muscle–transition zone. Only 13% failed in the middle part of the tendon and 26% failed at the enthesis/tendon–bone-insertion. In the groups with refixed tendons, the failure areas slightly shifted to the more distal parts with only 50% (CS–g–PCL group) and 45% (TGF–β3–CS–g–PCL group) in the proximal area, 42% (CS–g–PCL group) and 18% (TGF–β3–CS–g–PCL group) in the middle part, and 8% (CS–g–PCL group) and 36% (TGF–β3–CS–g–PCL group) in the area of the enthesis/tendon–bone-insertion, respectively. However, these differences were statistically not significant.

### 2.4. Tendon Healing in Rats: Histology

**Figure 3:**
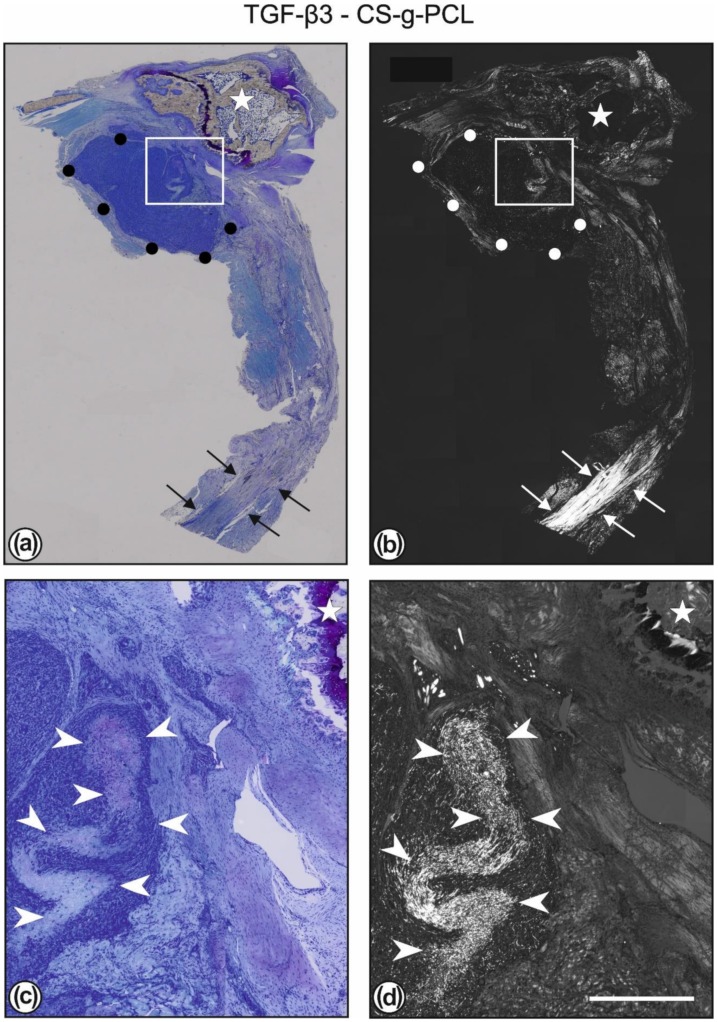
Toluidine blue staining (**a**) and polarisation optics (**b**) of the same section of a left shoulder with an implanted TGF–β3–CS–g–PCL scaffold. The white rectangles in (**a**,**b**) are shown in higher magnification in (**c**,**d**). The whole construct consists of humerus bone (white star), scaffold (outlined by black or white dots), and the reattached tendon. In (**b**) it can be clearly seen that a healthy and normal tendon (marked by white arrows) is present only at some distance away from the surgical site. The area between consists mostly of newly formed granulation tissue and degraded original muscle and tendon tissue. In (**c**,**d**) the granulation tissue ensheathing the TGF–β3–CS–g–PCL scaffold is shown. The infiltration of the scaffold with numerous cells can be seen, also the presence of areas with a still higher density of fibres and with only a few cells inside (marked by white arrowheads). Bar = 2000 µm (**a**,**b**); 400 µm (**c**,**d**).

*Toluidine blue stained sections* revealed the CS–g–PCL fibre scaffolds as well as the TGF–β3–CS–g–PCL fibre scaffolds showed good tissue integration. The scaffolds were embedded in granulation tissue including degenerated tendon tissue and muscles. Some blood vessels could be found in the surrounding tissue of the implant. Only minor fibrous structured tissue could be detected in the implant surrounding (Figure 3).

**Figure 4:**
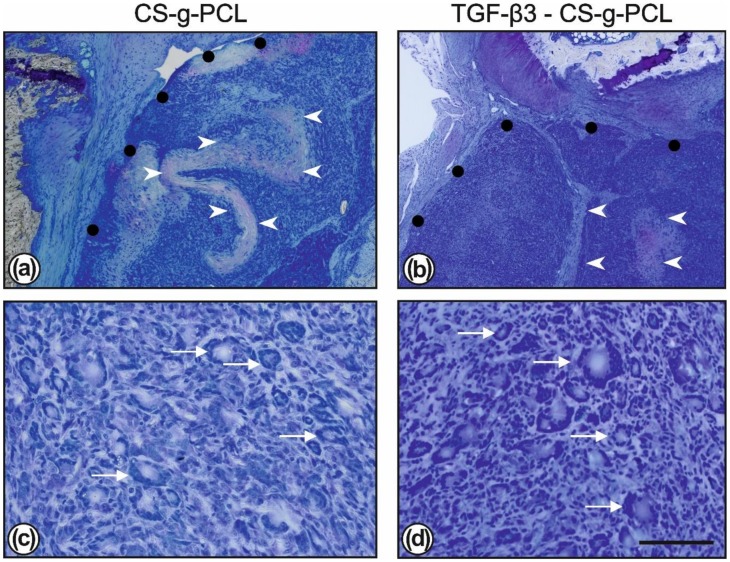
Toluidine blue staining showed the degradation of the CS–g–PCL scaffolds (outlined with black dots; (**a**,**c**)) and the TGF–β3–CS–g–PCL fibre scaffolds (outlined with black dots; (**b**,**d**)). The scaffolds are infiltrated with numerous cells; only a few areas with a denser fibrous structure are less infiltrated (marked by white arrowheads). At higher magnification the normal immune reaction is also indicated by the presence of numerous multinucleated foreign body giant cells (marked by white stars; c and d) which eliminate the degradation products of the scaffolds. Bar = 400 µm (**a**,**c**); 50 µm (**c**,**d**).

Both TGF–β3–CS–g–PCL and CS–g–PCL fibre scaffolds were infiltrated and enclosed with cells and many polynucleated foreign body giant cells were visible (Figure 4).

**Figure 5:**
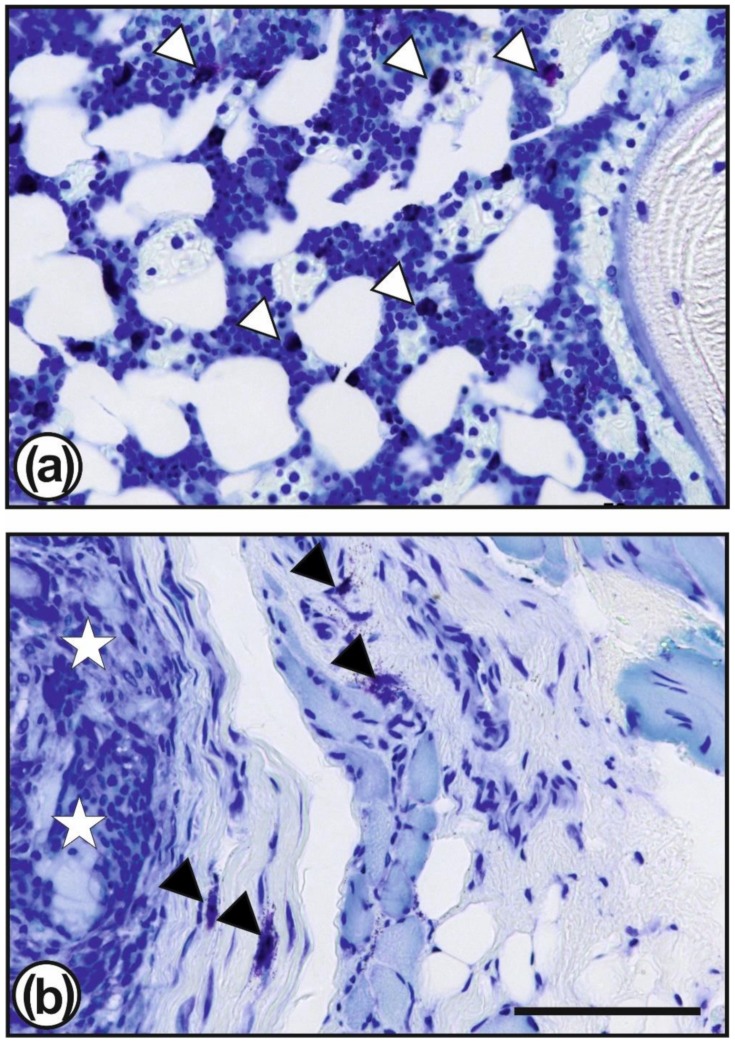
Toluidine blue staining showed mast cells (some of them are marked by white or black triangles) which are present numerously in the vicinity of the surgical site, especially in the bone marrow (**a**) but also in the granulation tissue surrounding the TGF–β3–CS–g–PCL fibre scaffolds (**b**; scaffold is marked by white stars). Bar = 50 µm.

In both groups, the PCL fibre matrix throughout the scaffold area was disintegrated, remainders in form of smaller sections of fibres could be found even after eight weeks. Seldom, small areas with a better preserved fibre matrix, i.e., with longer fibres and less infiltrated cells were observable (Figure 3c,d). Around the scaffolds some areas of undifferentiated granulation tissue were found, which were infiltrated with mast cells (Figure 5b). Mast cells were also found in the bone marrow (Figure 5a).

**Figure 6:**
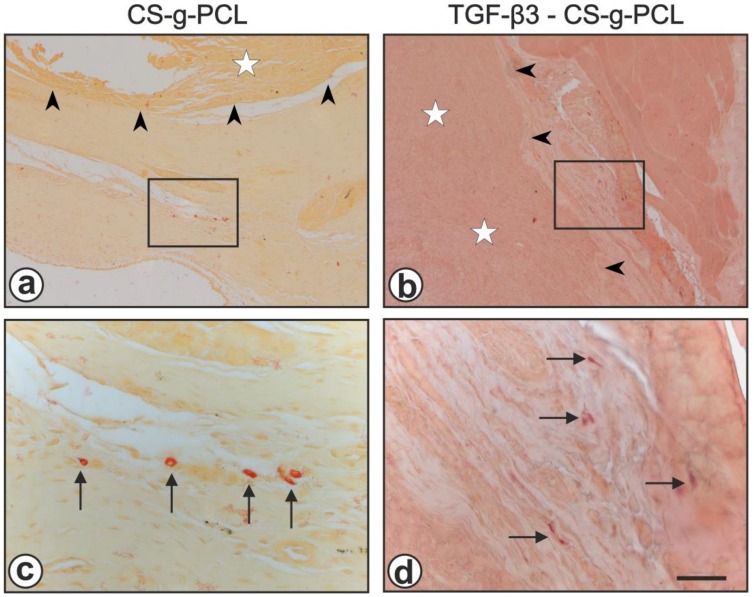
Chloroacetate esterase staining showed the absence of an acute inflammation, both in the CS-g-PCL group (**a**,**c**) and the TGF–β3–CS–g–PCL—group (**b**,**d**). The black rectangles in (**a**,**b**) are shown in higher magnification in (**c**,**d**). The boundary of the scaffolds is indicated by black arrowheads, the scaffolds are marked by white stars and chloroacetate esterase positive neutrophilic granulocytes are marked by black arrows. Bar = 200 µm (**a**,**b**); 50 µm (**c**,**d**).

In *chloroacetate esterase stained sections* in both groups no obvious acute inflammation was observed eight weeks after the 2nd surgery, only a few neutrophilic granulocytes in the vicinity of both the CS-g-PCL- and the TGF–β3–CS–g–PCL—scaffolds could be detected (Figure 6).

## 3. Discussion

In the present study, the effect of TGF–β3 loaded CS–g–PCL fibre scaffolds compared to unloaded CS–g–PCL fibre scaffolds was examined in a chronic rotator cuff tear rat model. TGF-β3 was chosen due to its reported effects on tendon healing processes [41,42,43]. In the present study, the TGF–β3–loaded fibre scaffolds showed a slightly higher stability of the tendon–bone constructs with respect to ultimate load to failure. Similar observations were also found by Manning and coworkers [42] who delivered TGF–β3 using a fibrin matrix in a rat model. They found an accelerated healing process and an improvement in structural properties after 28 days. Also in a rat model, Arimura and coworkers [45] observed significantly higher values for ultimate load to failure using TGF–β3 and TGF–β1, respectively. Arimura and coworkers used an acute tendon defect model with a dose of 100 ng TGF–β1 embedded in a hydrogel. They found a significant higher ultimate load to failure in animals treated with TGF–β1 hydrogels compared to animals treated with NaCl–soaked hydrogels, although tenogenic markers such as scx, Tnmd, Sox9, Coll1α1, and Coll3α1 were not significantly different between both groups until week eight. However they could show a decreased MMP9 and MMP13 expression two weeks postoperatively in the TGF–β1 treated group. Other authors could observe enhanced chondrogenesis after subcutaneous implantation of PDLLA–PEG hydrogel combined with mesenchymal stem cells and TGF–β3 [40] when compared to hydrogels without TGF–β3. In an in vitro study, mesenchymal stromal cells seeded onto decellularised equine superficial digital flexor tendon scaffolds with absorbed TGF–β3 showed elongated cell shapes, scaffold contraction and a musculoskeletal gene expression with an up-regulation of tenascin c and down-regulation of matrix molecules [46]. However, there are also studies which could not find any positive effect of TGF–β3 although TGF– β 3 was delivered with an osmotic pump throughout the complete observation period of four weeks [47]. These different findings might be caused by the observation that the repair processes during tendon healing seem to follow other paths than during normal developmental or true regeneration processes [48] and that in contrast to humans, rats have a remarkable capacity for spontaneous healing and regeneration of the rotator cuff [49].

Histologically no obvious differences between the two fibre scaffold variants could be observed after the follow up period. In both groups, disintegration of the implanted fibre scaffolds was far advanced. The original dense mesh-like structure had largely dissolved but fragments of the original long fibres were still present. Scattered mast cells were visible in the granulation tissue surrounding the original scaffold. Tang et al. reported on the role of mast cells as mediators of acute inflammatory reactions to implanted biomaterials [50] and showed that mast cells are necessary for the recruitment of macrophages and neutrophils. Although mast cell occurrence in the present study might have been a sign for an inflammatory response to the CS-g-PCL scaffolds (loaded and non-loaded), chloroacetate esterase staining could not show elevated levels of neutrophils. On the other hand macrophages and strikingly, a great number of foreign body giant cells were present within the original scaffold space. In the context of Tang et al. [50] these findings lead to the conclusion that mast cells mediated particularly the phagocytosis and therewith degradation of the implanted scaffolds rather than an inflammatory response. The high amount of foreign body giant cells is in contrast to results of other studies, where PCL was used as an implant material. In a rat dorsal pocket model where the medical grade PCL Osteomesh^®^ was used with an observation period of six weeks, no foreign body giant cell reaction occurred and only a few macrophages could be found [51]. A progressive encapsulation but also no foreign body giant cells could be found in a sheep cervical spine interbody fusion model using a magnesium–PCL cage [52]. However, it is known that physical features, such as the size or topography of the implant, as well as chemical features, such as the material characteristics or surface chemistry produce microenvironmental cues that modulate the response of the immune system [53]. The occurrence of foreign body giant cells is especially related to the size of the particles. When the particles (between 10 and 100 µm in diameter) cannot be phagocytosed by single macrophages, the macrophages fuse to form foreign body giant cells which try to remove the particles via internalisation or/and via extracellular digestion [54]. From an immunological point of view, fibres, i.e., elongated particles pose special challenges to the immune system. Short fibres are more readily internalised and play a minor role in inflammation processes whereas longer fibres lead to frustrated phagocytosis [55,56] provoking a chronic inflammatory activation. With respect to asbestos fibres it is known that longer fibres (> 5 µm) have a much higher fibrogenicity and carcinogenicity than shorter fibres [57]. Apart from fibre length, their diameter has also a distinct influence; intense foreign body giant cell reactions could also be observed in different rat models when electrospun polyethylene (PET) and PET/chitosan fibre meshes with a fibre diameter of less than 1 µm were used in an abdominal hernia model [58] and when PCL meshes as an outer shell to encase an inner porous chitosan core in a composite nerve graft were used [59] as well as in a subcutaneous air pouch mouse model when chitosan scaffolds with different acetylation degrees were tested [60]. From this point of view, it would be advantageous to use scaffolds with a larger fibre diameter to reduce these adverse effects. However, larger fibre diameter would also influence negatively the loading capacity of substances e.g., growth factors. Therefore, the development of resorbable scaffolds designed for the deposition of substances and the ingrowth of cells will have to find the optimal balance between both aspects. Besides this, the immune reaction is further determined by the relationship between the surface area of the biomaterial and the volume of the whole implant. High surface-to-volume implants such as mesh-like or porous scaffolds recruit higher amounts of macrophages and foreign body giant cells than do implants with smoother surfaces [60,61]. Concomitantly, the fibrous capsule ensheathing the implant is often found to be thinner on a porous surface than that on a dense and solid surface [62]. This is in line with our observations. In this study, a fibrous capsule was not present in both experimental groups, whereas in another study using a solid magnesium–PCL cage with a smooth surface as cervical spine spacer in a sheep model, a thick fibrous capsule was observed [52]. It is obvious that the complex interactions between wound healing and tissue regeneration which are necessary for successful implant integration highly depend upon the orchestrated and balanced response of the immune system.

Taken our findings together, TGF–β3 seems to have an effect on tendon healing, although the mechanisms remain incompletely understood. A limitation of the present study is the lack of early post-operative time points. It might be possible that obvious reactions to the CS–g–PCL biomaterial, which are predominantly expressed as foreign body giant cell reactions, mask origin effects of TGF–β3. Additionally, the release kinetics of TGF–β3 in vivo is not known and it is uncertain if the local concentration of TGF–β3 at the surgical site and in an inflammatory environment is high enough to initiate a biological effect. Furthermore, methods to deeper specify the immunological tissue reactions could not be provided and should be implemented in future studies. Especially macrophage differentiation is important to evaluate their presence, because early activated M1 macrophages produce proinflammatory cytokines and inducible nitric oxide synthase (iNOS) while later activated M2 macrophages induce proregenerative processes and therewith would lead to a different assessment.

In conclusion, TGF–β3 loading of electrospun CS–g–PCL fibre scaffolds showed advantages in biomechanical stability of the repair site compared to unloaded CS–g–PCL fibre scaffolds—but not at the histomorphological level. Foreign body giant cells were the dominant cell type during the degradation process of the PCL fibre scaffolds in both groups. Although it cannot be definitely stated if they have positive or negative influences on the healing process it is assessed as an adverse reaction to a biomaterial, at least in the extend which was observed in the present study. Further investigations are necessary to characterize their polarisation and their general impact on tendon healing.

## 4. Materials and Methods

### 4.1. CS–g–PCL Coated Fibre Scaffold

The fibre scaffolds were produced by electrospinning of poly–ε–caprolactone (PCL; M_n_ = 80,000 g/mol, Sigma-Aldrich Chemie, Taufkirchen, Germany) solved in trifluoroethanol (abcr, Karlsruhe, Germany) as described in de Cassan and coworkers [63]. The fibre scaffolds exhibited a mean fibre diameter of 1.61 ± 0.97 µm (as determined by the analysis of scanning electron microscopy pictures) and the uniaxial tensile testing resulted in mean force at failure of 27.59 ± 10.1 N and a mean elongation at failure of 104.32 ± 13.18% (as determined by using a uniaxial testing machine; for details see [44]. These scaffolds were then modified using CS–g–PCL (in detail described in [32] which was bound to the PCL fibre surface by crystallization according to established methods [64].

### 4.2. Sterilisation

The CS–g–PCL fibre scaffolds were cut into pieces of 3 mm × 5 mm, shrink-wrapped in sterilisation pouches (SteriClin, Vereinigte Papierwarenfabriken, Feuchtwangen, Germany) and sterilised using a Rhodotron TT100 e–beam accelerator (25 kGy; Mediscan, Kremsmünster, Austria). The used sterilisation process does not have any influence on material properties [65].

### 4.3. Loading of the CS–g–PCL Coated PCL Fibre Scaffolds With TGF–β3

The loading of the CS–g–PCL coated PCL fibre scaffolds with TGF–β3 was performed under sterile conditions [66]. Briefly, TGF–β3 was encapsulated into chitosan–tripolyphosphate nanoparticles and loaded onto the fibre scaffolds for subsequent release. For preparation of the nanoparticles, chitosan was purified according to an established protocol [67]. Purified chitosan with an acetylation degree of 42% (DA 42%) was dissolved in 0.1% (*v/v*) acetic acid to a concentration of 1 mg/mL. TGF–β3 (from *Escherichia coli*, Peprotech, Hamburg, Germany; dissolved in PBS + 0.1% (w/v) bovine serum albumine; stock concentration 10 µg/mL) was carefully mixed with this solution. Tripolyphosphate was dissolved in Millipore water to obtain a concentration of 1 mg/mL which was rapidly mixed with the chitosan–TGF–β3 solution in a 1:3 ratio resulting in TGF–β3–loaded chitosan–tripolyphosphate nanoparticles. The modified fibre scaffolds (PCL modified with CS–g–PCL plus alginate) were incubated in this solution for 15 min. Therewith a TGF–β3 loading capacity of 250 ng/scaffold was reached, which is in line with different in vitro studies (e.g., [46,68]). Before further use, the fibre scaffolds were washed twice for one minute with Millipore water. All chemicals, if not otherwise stated, were purchased from Sigma-Aldrich.

### 4.4. Animal Study

The study was approved by the local institutional animal care and research advisory committee and permitted by the competent authority (Niedersächsisches Landesamt für Verbraucherschutz und Lebensmittelsicherheit; reference number 33.12–42502–04–15/2015) 29 December 2015 of the federal state Niedersachsen, Germany. Male Lewis rats (*n* = 17, mean weight: 482 g ± 31.52 g; minimum age: 16 weeks; strain: Lew/Ztm, Zentrales Tierlabor; Hannover Medical School) were housed in pairs in standard cages (UNO Type IVS, UNO BV, Zevenaar, The Netherlands) with food (maintenance diet for rats/mice, Altromin International, Lage, Germany) and water ad libitum and an adaption time of at least 14 days. Anaesthesia was initiated by a mixture of isoflurane (5 vol%) and oxygen and maintained by isoflurane (2–3 vol%; Isofluran CP, cp–pharma, Burgdorf, Germany) in an oxygen mixture using a breathing mask. Rimadyl^®^ (Carprofen, 5 mg/kg, Zoetis, Berlin, Germany) and Butorgesic^®^ (Butorphanol, 0.01–0.05 mg/kg, cp–pharma) were used as analgesia before surgery.

The whole experimental procedure was designed according to an earlier performed study [44] and consisted of two independent surgical interventions with an interval of four weeks. In the first surgery, a chronic tendon tear defect was initiated. To this end, the skin was clipped and disinfected in the left shoulder area, an incision was made and the deltoid muscle was split. Then, the proximal part of the musculus triceps brachii (caput laterale) was incised to reach the tendon insertion at the humeral tuberculum majus. There, the infraspinatus tendon was detached and wrapped in Dura–Guard^®^ (Baxter Healthcare, Glattpark, Switzerland) to encase the tendon stump and thus prevent the spontaneous reattachment and healing of the tendon. The Dura–Guard^®^ cover was fixed with two single sutures (Mersilk, 6.0, Ethicon LLC, Livingston, Scotland) and wound closure was performed layer by layer using Vicryl^®^ 4–0 (Ethicon). After surgery, the rats were treated with antibiotics (Baytril^®^, Enrofloxacin 5 mg/kg, Bayer, Leverkusen, Germany, for 3 days) and analgesics (Rimadyl^®^, Carprofen 5 mg/kg, Zoetis Schweiz, Delémont, Switzerland) for the following 2 to 8 days depending on the individual score values (see below).

Four weeks later in the second surgery, the tendon, and a PCL fibre scaffold coated with TGF–β3 were surgically reattached to the bone. After opening of the surgical site, first the Dura–Guard^®^ wrapping was completely removed from the tendon stump. Then, two crossing channels were drilled into the tuberculum majus with a drill bit (RIS.590.201, Ø 0.45 mm, RISystem AG, Landquart, Switzerland) to provide an anchoring option for the surgical sutures. In the following step, the tendons with the overlaid PCL fibre scaffold coated with TGF–β3 was refixed using resorbable suture material (Vicryl^®^ 4–0, Ethicon, Johnson & Johnson Medical GmbH, Norderstedt, Germany); for a detailed description see [44]. The incised muscles were adapted and fascia and skin were closed in two layers. After surgery, the rats were treated with antibiotics and analgesics as described above.

The individual score values were collected daily in the first two weeks after each surgery and the observations were scored with respect to “general condition”, “spontaneous behaviour”, “wound assessment”, and “weight loss“ (0–20 points each; 0 = the normal value, 20 = highly altered), and “lameness” (0–10 points) respectively. Lameness as main evaluation parameter was subdivided in normal gait (score 0), slight (score 1) and moderate (score 2) deviation from the normal gait, abnormal standing position (score 5) or no weight bearing on the operated limb (score 10). Weight loss was classified in less than 5% (score 1), 5–10% (score 2), 11–20% (score 10) and more than 20% (score 20). Eight weeks after the second surgery, the animals were finally anaesthetised and sacrificed and the respective parts of the rotator cuff were analysed biomechanically (11 animals, one drop out) and histologically (5 animals). The respective parts of the unaffected contralateral right rotator cuff served as intact native control.

Due to ethical reasons and legal limitations and to reduce the number of animals to a reasonable limit, the comparison group (CS-g-PCL group) was taken from an identical conducted study [44].

### 4.5. Biomechanical Testing

For biomechanical testing, the whole construct (tendon–enthesis–humerus) was carefully removed and dissected from remaining muscles and connective tissue. This was possible without any problems in case of the intact right tendons but the left tendons were ensheathed by a tissue encapsulation which was mostly left untouched to avoid any mechanical lesion of the construct. For testing, the humerus was first placed in a cylindrical block of polymethyl methacrylate (Technovit^®^ 4004, Heraeus–Kulzer, Hanau, Germany) in an angle of about 30° relative to the vertically oriented tendon to mimic the anatomical and physiological situation [44,69]. Then, the free end of the infraspinatus tendon was clamped with pieces of fine blotting paper between two halves of a modified and custom-designed split dumbbell clamp and fixation device, including a stabilising fork-like outrigger, according to Duchman and coworkers [70]. The specimens were mounted into a static materials testing machine (Zwick 1445, Zwick, Ulm, Germany) and preloaded with 0.25 N. After holding the preload for five seconds, the specimens were preconditioned with 3% strain at 1 Hz for five cycles. After that, specimens were loaded to failure with 20 mm/s and the acting force up to the final force at failure [N] was recorded. Resulting values were analysed statistically.

### 4.6. Embedding in Technovit^®^ 9100 New

For histology, the removed tissues were fixed in 3.7% buffered commercial formalin (Otto Fischar, Saarbrücken, Germany) for two days at 4 °C. Then, the specimen were embedded and polymerised in methyl methacrylate (Technovit^®^ 9100 New, Heraeus–Kulzer, Hanau, Germany) according to the manufacturer’s instructions and established protocols [71]. After polymerisation, the tissue blocks were cut into 5 µm thin longitudinal sections using a RM 2155 microtome and tungsten carbide knives (Leica, Bensheim, Germany). The sections were placed onto poly–L–lysine–coated glass slides and stretched with 90% ethanol to remove wrinkles. The slides were pressed in a clamp box and dried for seven days at 37 °C. Prior to staining, the sections were deacrylated in xylene (2 × 10 min) and 2–methoxyethylacetate (1 × 10 min) and rehydrated with a decreasing alcohol series.

### 4.7. Toluidine Blue Staining

Deacrylated and rehydrated sections were incubated in 0.1% Toluidine blue O (Sigma-Aldrich Chemie, Taufkirchen, Germany) for 40 s, washed in distilled water, dehydrated in alcohol, and mounted with Eukitt (Labonord, Mönchengladbach, Germany).

### 4.8. Chloroacetate Esterase Staining

For the detection of neutrophilic granulocytes, chloroacetate esterase staining was used. Deacrylated and rehydrated sections were first washed in distilled water and then incubated with naphthol AS-D chloroacetate (Sigma-Aldrich) in 4% pararosaniline (Chroma, Olching, Germany) and 4% sodium nitrite in 0.1 M acetate buffer for 120 min at 37 °C. Sections were washed in distilled water and mounted with Aquatex (Merck, Darmstadt, Germany). Cells containing red-brownish granules were regarded as positive. Control sections were incubated without the substrate. No staining developed in these controls.

### 4.9. Microscopy

Photomicrographs were taken with an Axioskop 40 microscope equipped with bright field and polarisation optics, an AxioCam Mrc digital camera and AxioVision software (all from Carl Zeiss, Oberkochen, Germany). Evaluation was performed descriptively.

### 4.10. Statistical Analysis

The statistical analysis regarding the differences between the three groups was carried out with a nonparametric statistical test (Kruskal–Wallis test) for nonrelated samples using SPSS (SPSS Statistics 24, IBM, Ehningen, Germany). A significance level of α = 0.05 was applied.

## Abbreviations

TGF	Transforming Growth Factor
CS–g–PCL	chitosan coated polycaprolacton
PCL	chitosan coated polycaprolacton
Scx	Scleraxis
Tnmd	Tenomodulin
Sox9	Transcription factor SOX-9
Coll1α1	Collagen 1α1
Coll3α1	Collagen 3α1
MMP9	Matrix metallopeptidase 9
MMP13	Matrix metallopeptidase 13
